# A Meta-Analysis Including Pre-selected Sequence Variants Associated With Seven Traits in Three French Dairy Cattle Populations

**DOI:** 10.3389/fgene.2018.00522

**Published:** 2018-11-06

**Authors:** Andrew G. Marete, Bernt Guldbrandtsen, Mogens S. Lund, Sébastien Fritz, Goutam Sahana, Didier Boichard

**Affiliations:** ^1^UMR GABI, INRA, AgroParisTech, Université Paris Saclay, 78350 Jouy en Josas, France; ^2^Center for Quantitative Genetics and Genomics, Aarhus University, Aarhus, Denmark; ^3^ALLICE, Paris, France

**Keywords:** candidate genes, candidate SNP, GWAS, meta-analysis, sequence imputation

## Abstract

A within-breed genome-wide association study (GWAS) is useful when identifying the QTL that segregates in a breed. However, an across-breed meta-analysis can be used to increase the power of identification and precise localization of QTL that segregate in multiple breeds. Precise localization will allow including QTL information from other breeds in genomic prediction due to the persistence of the linkage phase between the causal variant and the marker. This study aimed to identify and confirm QTL detected in within-breed GWAS through a meta-analysis in three French dairy cattle breeds. A set of sequence variants selected based on their functional annotations were imputed into 50 k genotypes for 46,732 Holstein, 20,096 Montbeliarde, and 11,944 Normande cows to identify QTL for milk production, the success rate at insemination of cows (fertility) and stature. We conducted within-breed GWAS followed by across-breed meta-analysis using a weighted Z-scores model on the GWAS summary data (i.e., *P*-values, effect direction, and sample size). After Bonferroni correction, the GWAS result identified 21,956 significantly associated SNP (*P*_FWER_ < 0.05), while meta-analysis result identified 9,604 significant SNP (*P*_FWER_ < 0.05) associated with the phenotypes. The meta-analysis identified 36 QTL for milk yield, 48 QTL for fat yield and percentage, 29 QTL for protein yield and percentage, 13 QTL for fertility, and 16 QTL for stature. Some of these QTL were not significant in the within-breed GWAS. Some previously identified causal variants were confirmed, e.g., BTA14:1802265 (fat percentage, *P* = 1.5 × 10^−760^; protein percentage, *P* = 7.61 × 10^−348^) both mapping the DGAT1-K232A mutation and BTA14:25006125 (*P* = 8.58 × 10^−140^) mapping *PLAG1* gene was confirmed for stature in Montbeliarde. New QTL lead SNP shared between breeds included the intronic variant rs109205829 (*NFIB* gene), and the intergenic variant rs41592357 (1.38 Mb upstream of the *CNTN6* gene and 0.65 Mb downstream of the *CNTN4* gene). Rs110425867 (*ZFAT* gene) was the top variant associated with fertility, and new QTL lead SNP included rs109483390 (0.1 Mb upstream of the *TNFAIP3* gene and 0.07 Mb downstream of *PERP* gene), and rs42412333 (0.45 Mb downstream of the *RPL10L* gene). An across-breed meta-analysis had greater power to detect QTL as opposed to a within breed GWAS. The QTL detected here can be incorporated in routine genomic predictions.

## Introduction

Ideally, the magnitude of estimated effects on the quantitative trait of interest could be used to rank single nucleotide polymorphisms (SNP) for a functional genomic study to identify causal variants. The physical locations of the associated SNP on the genome are then flagged and mined for causative variants underlying quantitative trait loci (QTL). Causal variants reported for QTL in previous dairy cattle studies include polymorphisms causing variation in milk production, fertility, and embryo mortality (Grisart et al., 2002; Hoff et al., 2017; Michot et al., 2017; Bouwman et al., 2018). Most analyses testing genomic regions, like those above, perform within-breed GWAS (Chang et al., 2018). Even so, our knowledge regarding causal variants in these genomic regions remains limited because the GWAS analyses yield similar *P*-values for many adjacent SNP variants, a consequence of linkage disequilibrium (LD). Even with a multitude of GWAS results, strong LD prevents distinguishing the actual causal variant from linked markers (Goddard and Hayes, 2009). A meta-analysis can be used to improve the resolution of QTL detection and identify causal variants provided that LD is conserved at short distances across breeds (van den Berg et al., 2016). The main advantage of a meta-analysis is that it allows simultaneous analysis of many breeds by combining GWAS summary statistics across populations, thereby increasing power to detect QTL (van den Berg et al., 2016; Bouwman et al., 2018).

In this paper, we report an across-breed meta-analysis of GWAS summary statistics based on imputed pre-selected sequence variant genotypes from 78,772 cattle from three dairy breeds and for seven traits. The meta-analysis across breeds allowed us to identify 142 QTL for milk production, stature, and fertility.

## Materials and methods

### Studied population and phenotypes

We studied phenotypes from three French dairy cattle breeds. No ethical approval was necessary since the data used was from existing databases. Phenotypes were from cows born between 2007 and 2013 in the French dairy production regions and were defined as follows: (1) Production traits including milk, protein, and fat yields were obtained from test-day records expressed as 305 d yield (kg). Fat content (g/kg) was calculated as 1,000^*^(fat yield/milk yield). Protein content (g/kg) was calculated as 1,000^*^ (protein yield/milk yield). (2) Fertility was defined as success/failure at each insemination of lactating cows and recorded as a success (= 1) or a failure (= 0). (3) Stature was measured as the vertical distance from the plank to the sacrum.

These traits were analyzed using the French national evaluation model (Boichard et al., 2012b) to obtain Yield Deviations (YD) (VanRaden and Wiggans, 1991). A YD can be defined as the mean performance adjusted for all environmental effects, including the permanent environment for cows with multiple records. The model varied according to the trait, especially concerning environmental factors. We retained traits for genotyped cows and this included data from 46,732 Holstein cows, 20,096 Montbeliarde cows, and 11,944 Normande cows.

### Genotyping and imputation

**Figure 1:**
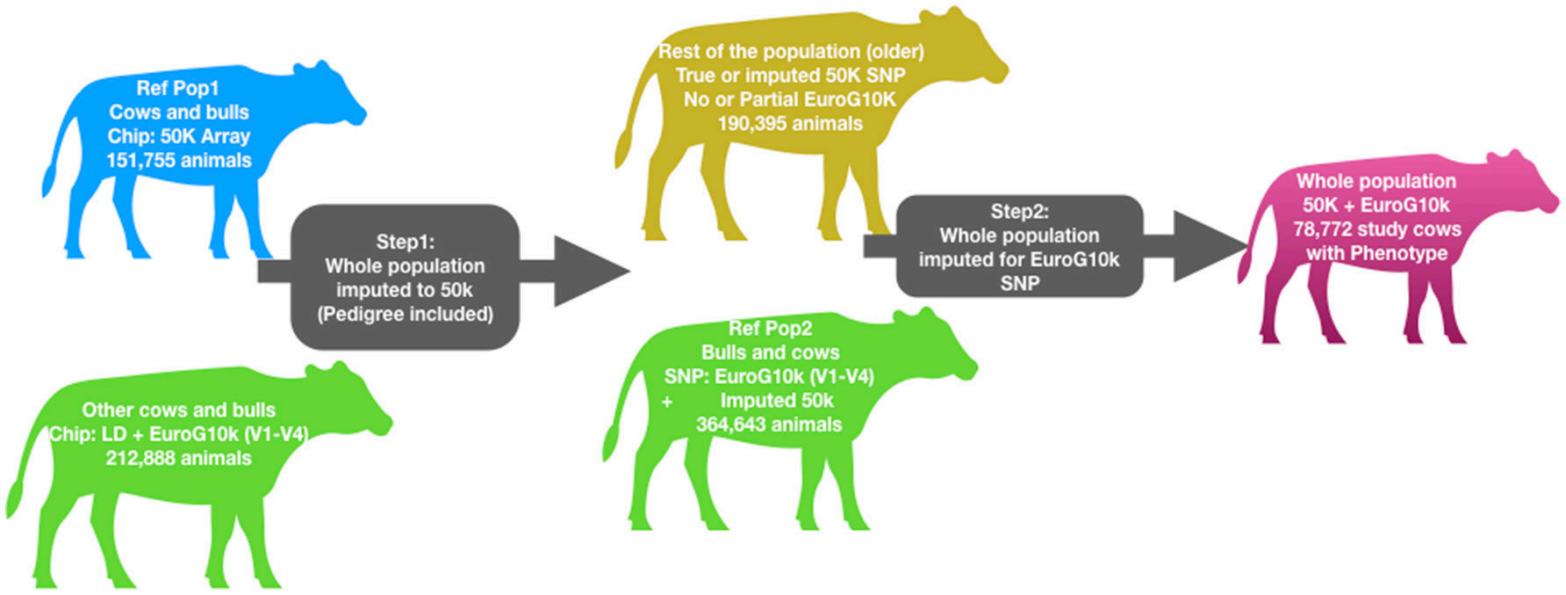
Genotype imputation workflow. Table showing number of animals genotyped with various chips is included in Supplementary Table 1.

Cows were genotyped using the Illumina Infinium® BovineSNP50 BeadChip (50 K, Illumina, San Diego, CA), BovineLD BeadChip (Boichard et al., 2012a) or one of the first four versions of the EuroG10k SNP chip (Boichard et al., 2018). The EuroG10k SNP chip is composed of two parts: (1) Between 7,000 and 8,500 generic (and supposedly neutral) SNP from BovineLD Genotyping BeadChip v.2 (Boichard et al., 2012a). (2) Whole genome sequence variants-a custom part of up to 7,232 SNP selected from sequence data as part of 1,000 Bull Genomes Project *Run 4* (Daetwyler et al., 2014) based on their functional annotation. The full description of the EuroG10k chip and quality control procedure was previously described by Marete et al. (2018a) and Boichard et al. (2018). Cows with phenotypes were rather old animals and were genotyped with the 50 k chip. Younger animals including cows without phenotypes were genotyped with the EuroG10k chip. To get complete marker information across animals, and to include the sequence variants in the analysis, we run two successive steps of imputation for the entire populations (males and females, with and without phenotypes, see Figure 1). First, we imputed all 50 k markers for all animals using animals with 50 k genotypes as a reference. Then we imputed sequence variants using the animals genotyped with the EuroG10k chip as the reference population. The imputation was done within breed using the FImpute software (Sargolzaei et al., 2014). After imputation, 48,576 SNP distributed across 29 *Bos taurus* autosomes (BTA) remained. Of these, 42,967 were from the 50 k chip, and 5,609 sequence variants selected from French cattle populations. Imputation accuracy was estimated using both concordance rate and allelic squared correlation (*r*^2^), both for animals genotyped with 50 k only and those genotyped with EuroG10k chip only. In both instances, we randomly masked 20% of the genotyped markers on each chromosome and 15% of the cows per breed, then performed an imputation and compared the imputed genotypes with true genotypes by estimating a Pearson correlation coefficient. For the 50 k SNP, the average concordance was >0.98, and average allelic *r*^2^ was >0.97, and for the sequence variants, the average concordance was >0.95, and average *r*^2^ was >0.96 for all breeds.

### Association studies, meta-analysis, and QTL heterogeneity

First, 48,576 SNP (pre-selected sequence variants and 50 k SNP) were tested individually for association with each trait within the breed. The GCTA software (Yang et al., 2011) was used to fit a mixed linear regression model to test associations between a SNP and the trait. For any given trait, the fitted model was

(1) $$\begin{array}{r} \text{y}=1\mu +\;\text{x}\beta +\;\text{g}+\varepsilon \end{array}$$

where **y** was a vector of phenotypes (yield deviation) for all cows, **1** was a row vector of 1s, μ was the mean, ***x***was the vector of true or imputed allele dosages for the SNP, β was the fixed allele substitution effect of the SNP, **g** was the vector of random additive genetic effects with $\text{g}\sim N\left(0,\;{\text{G}\sigma }_{g}^{2}\right)$, **G** being the genomic relationship matrix, $\sigma_{g}^{2}$ was the variance explained by all the SNPs, ε was a vector of random residual effects with $\varepsilon \;\sim \;N\left(0,\;{\text{I}\sigma }_{\varepsilon }^{2}\right)$, $\sigma_{e}^{2}$ was the error variance, and **I** was an identity matrix. The variance of **y** was $var\left(\text{y}\right)=\;{\text{G}\sigma }_{g}^{2}+\;{\text{I}\sigma }_{\varepsilon }^{2}$. The *g*_*jk*_ term of **G** matrix was estimated by

(2) $$\begin{array}{r} g_{jk}=\;\frac{1}{w}\;\sum_{i=1}^{w\;}\frac{\left(z_{ij}-{2p}_{i}\right)\left(z_{ik}-\;{2p}_{i}\right)}{{2p}_{i}\left({1-p}_{i}\right)} \end{array}$$

where w was the total number of SNP, *z*_*ij*_*and z*_*ik*_ were numbers of copies of the reference allele for the *i*^*th*^ SNP in the *j*^*th*^ and *k*^*th*^ cows, respectively, and *p*_*i*_ was the frequency of the reference allele estimated from the marker data (VanRaden, 2008).

To control the type I error rate and assuming all tests were independent (a conservative assumption due to LD among SNP), a Bonferroni genome-wide correction was applied for the total number of tests for each trait i.e., the nominal type I error rate (α = 5%) was divided by the number of SNP (w = 48,576) to obtain a genome-wide error rate threshold of *t* = 1.03 × 10^−6^. Any SNP whose probability of observing the test statistics was below this value was considered genome-wide significant.

The within-breed analyses were followed by a meta-analysis using weighted Z-score model as implemented in METAL software (Willer et al., 2010). The weighted Z-score model used *P*-values and directions of effect estimates and weights individual GWAS based on the sample size to compute a Z-score, i.e.,

(3) $$\begin{array}{r} Z_{b}=\Phi^{-1}\;\left(1-\frac{P_{b}}{2}\right)*\Delta b \end{array}$$

where *Z*_*b*_ was the Z-score for breed *b*, Φ was the standard normal cumulative distribution, *P*_*b*_ was the *P*-value, Δ*b* was the direction of the SNP effect estimated within breed *b*. Overall Z-score was calculated as:

(4) $$\begin{array}{r} Z=\;\frac{\sum_{b}Z_{b}w_{b}}{\sqrt{\sum_{b}w_{b}^{2}}} \end{array}$$

where $w_{b}=\;\sqrt{N_{b}}$ and *N*_*b*_ was the sample size for breed *b*. The overall *P*-value was calculated as:

(5) $$\begin{array}{r} P=\;2\Phi \left(-|Z|\right) \end{array}$$

We evaluated heterogeneity of the effect sizes across breeds using Cochran's *Q*-test (Cochran, 1954) as implemented in the METAL software. A QTL was deemed to segregate across populations if the heterogeneity *P*-value was ≤ 0.05. The functional consequence of significantly associated variants was predicted using the Variant Effect Predictor tool from Ensembl Genome Browser 90 (McLaren et al., 2016). We classified variants that (i) mapped to a gene, (ii) < 5-kb to known genes, and, (iii) >5-kb from any coding region. In case of multiple variants representing the same gene, we kept the variant associated with most traits and with the lowest *P*-value as representative for that gene.

### Identification of QTL that segregates within and across breeds

Since it is challenging to identify the causative variant, we aimed at determining the QTL region that most likely harbor the causal variant. First, for each chromosome, we subjectively inspected significantly associated variants within the genomic regions. Secondly, we defined the within-breed QTL region as 1 Mb windows and the SNP with the lowest *P*-value designated as the lead SNP. A QTL was selected as significant in more than one breed if any of the SNP in the QTL region of the other breed had a *P*-value lower than the genome-wide threshold (*P* < 1.03 × 10^−6^). The UMD3.1 (Zimin et al., 2009) bovine genome annotation was used to annotate genes centering within 1 Mb intervals on the lead SNP. We compared these annotations to known QTL for bovine milk production, fertility and stature traits using QTLdb (Hu et al., 2016) and literature review.

## Results

### Association studies

**Figure 2:**
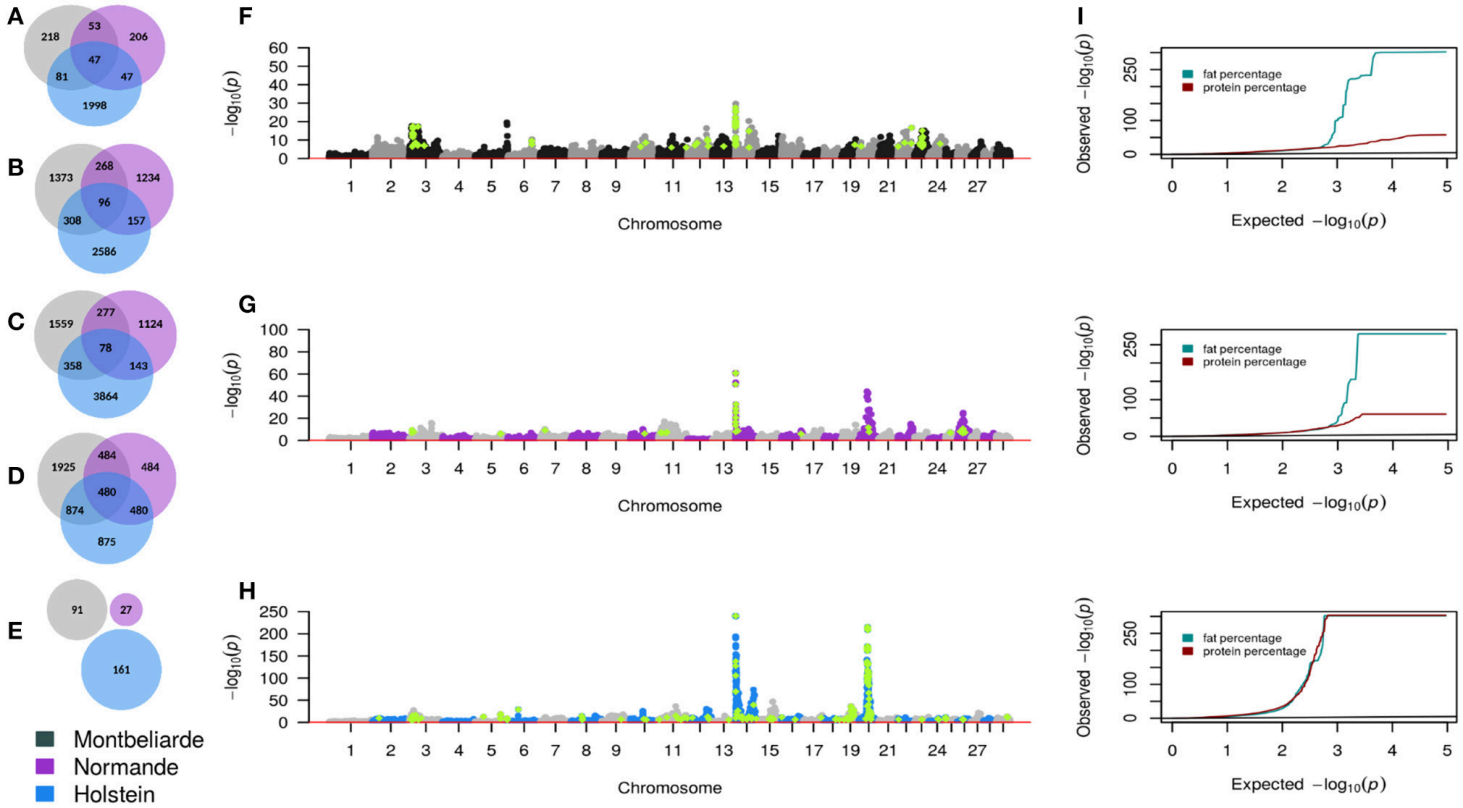
GWAS summary statistics. Venn diagrams representing significant SNP from GWAS for milk yield **(A)**, fat percentage **(B)**, protein percentage **(C)**, stature **(D)**, and fertility **(E)**. Composite Manhattan and corresponding quantile-quantile (QQ) plots **(I)** showing the association of imputed sequence variant with fat and protein percentage in Montbeliarde **(F)**, Normande **(G)**, and Holstein **(H)**. Each dot shows the most significant *P*-value that was observed across both traits. Light green color represents sequence variants with *P* < 1 × 10^−6^.

Overall, there were 21,956 SNP significantly associated with traits in three French cattle breeds. Forty-three percent of these associated SNP were from Holstein, 24% from Montbeliarde, 14% from Normande. Seven percent of these SNP showed association in both Holstein and Montbeliarde, 4% in Holstein and Normande, 5% in Montbeliarde and Holstein, and 3% in all three breeds. Production traits and stature had more significant hits across breeds, compared to fertility. As presented in Figures 2A–E, the overlap of significant tests in any two-breed combination and for all traits was most evident between Holstein and Montbeliarde (1,621 SNP) and least evident between Montbeliarde and Normande (1,082 SNP). Seven hundred and one SNP were significantly associated with the same trait in all three breeds.

As presented in Figures 2F–I, some of the imputed sequence variants had lower *P*-values compared with the 50 k variants *P*-values. Although more than 13% of the pre-selected sequence variants had MAF between 0.5 and 5%, only 2 QTL lead SNP had MAF lower than 5%. We did not detect QTL with MAF < 5% in Normande. Among the pre-selected sequence variants, 1,478 were significant (*P* < 1.03 × 10^−6^).

### Within breed GWAS and multi-breed meta-analysis

#### Production traits

##### Milk yield

We observed both known and novel QTL in all three cattle breeds. The most significant QTL lead SNP from meta-analysis are presented in Table 1. We observed QTL on 16 chromosomes where Holstein had 23 QTL, Montbeliarde had 10 QTL, Normande had 15 QTL. The most significant lead SNP was located at *Bos taurus autosome* (BTA) 14:1801116 (rs109421300, *P* = 9.7 × 10^−283^), an intronic variant near the causal variants in the *DGAT1* gene. Other significant QTL were observed at BTA5:50804085 (*P* = 1.3 × 10^−22^, *intergenic*), rs109205829 (BTA8:30088133, *P* = 2.3 × 10^−21^, *NFIB* gene), and BTA20:32670639 (*P* = 4.0 × 10^−41^, intergenic flanking 3′ end of the *GHR* gene).

**Table 1:** Lead SNP variants at 36 QTL for milk yield.

								**MON**		**NOR**		**HOL**	
**QTL**	**Reference SNP cluster ID**	**CHR**	**BP**	**Allele**	**Nearby gene**	**P_meta_**	**Effect direction (and evidence of heterogeneity) across three populations^1^**	**b(se)**	**p**	**b(se)**	**p**	**b(se)**	**p**
1	rs29015637	2	9810493	a/g	—	4.2e-09	−−−(4.7e-02)	−49.52(11.91)	3.2e-05	−1.91(13.46)	8.9e-01	−47.76(9.88)	1.4e-06
2	rs41615145	2	45026036	t/c	—	4.5e-17	+++(1.5e-02)	−23.33(13.31)	7.9e-02	−47.80(11.70)	4.4e-05	−50.94(6.62)	1.5e-14
3	rs42889305	2	70680985	t/g	—	1.8e-12	−−−(7.7e-05)	1.26(9.47)	8.9e-01	25.81(12.58)	4.0e-02	54.29(6.77)	1.0e-15
4	rs43709763	3	20031136	a/c	*CTSS*	3.0e-15	−−−(3.7e-06)	41.84(28.21)	1.4e-01	2.16(23.05)	9.3e-01	63.30(6.86)	2.9e-20
5	rs29023272	3	50804085	a/g	*RPL5*	1.3e-22	+++(5.9e-04)	−37.17(8.57)	1.4e-05	−6.29(10.69)	5.6e-01	−72.86(7.62)	1.1e-21
6	rs42737927	3	113907407	t/c	*UGT1A1*	1.3e-11	+++(6.5e-03)	−21.99(9.70)	2.3e-02	−3.71(10.06)	7.1e-01	−49.75(7.00)	1.2e-12
7	rs109960400	7	19676040	t/c	*RANBP3*	4.8e-08	+++(8.2e-02)	−18.69(10.88)	8.6e-02	−23.29(32.57)	4.7e-01	−52.12(9.31)	2.2e-08
8	rs110220494	7	46109256	a/g	*AFF4*	3.9e-10	+++(2.2e-02)	287.35(326.21)	3.8e-01	25.24(10.79)	1.9e-02	42.47(6.67)	2.0e-10
9	rs109939693	7	59590759	t/c	—	8.6e-12	−−−(4.8e-01)	−64.57(21.74)	3.0e-03	−18.78(10.27)	6.7e-02	−46.45(7.75)	2.1e-09
10	rs109205829	8	30088133	c/g	*NFIB*	2.3e-21	+++(5.4e-01)	36.55(9.17)	6.8e-05	46.35(10.43)	8.8e-06	51.51(6.90)	8.4e-14
11	rs42254183	8	65107050	a/g	—	2.8e-15	+++(9.5e-05)	−6.83(12.47)	5.8e-01	−39.07(15.38)	1.1e-02	−83.55(9.70)	7.3e-18
12	rs134292120	8	102635397	a/t	*ZNF483*	6.3e-13	−−−(4.3e-03)	−21.66(16.11)	1.8e-01	−149.15(90.16)	9.8e-02	−82.17(10.78)	2.5e-14
13	rs41612115	9	51985736	t/c	—	9.1e-19	+++(2.3e-01)	46.39(10.67)	1.4e-05	27.06(13.88)	5.1e-02	68.45(8.95)	2.1e-14
14	rs110315613	10	13737253	t/c	—	5.5e-12	+-+(4.5e-05)	−25.23(13.52)	6.2e-02	6.12(11.88)	6.1e-01	−74.79(9.36)	1.4e-15
15	rs110053896	10	50918709	a/g	*FAM81A*	2.1e-15	+++(1.1e-03)	−72.30(37.69)	5.5e-02	−15.77(13.71)	2.5e-01	−61.91(7.32)	2.7e-17
16	rs109750969	10	91086389	a/g	—	1.3e-15	−−−(9.6e-05)	15.26(9.43)	1.1e-01	17.52(20.81)	4.0e-01	68.24(7.67)	5.9e-19
17	rs109209415	11	101301047	t/c	*LAMC3*	2.9e-15	+++(5.8e-01)	−79.37(25.50)	1.9e-03	−46.84(15.26)	2.1e-03	−48.71(7.31)	2.7e-11
18	rs110434838	13	40837170	a/g	*KIZ*	9.3e-18	−−−(7.1e-01)	59.02(14.04)	2.6e-05	106.10(40.14)	8.2e-03	75.29(10.68)	1.8e-12
19	rs109421300	14	1801116	t/c	*DGAT1*	9.7e-283	+++(7.7e-43)	−370.01(45.63)	5.1e-16	−177.48(18.09)	1.0e-22	−314.84(8.66)	1.1e-289
20	rs41657812	14	5952697	a/g	—	1.2e-29	−−−(9.5e-17)	3.83(8.75)	6.6e-01	4.44(10.91)	6.8e-01	94.83(6.68)	1.1e-45
21	rs41629306	14	40812291	a/c	*HNF4G*	1.2e-16	+++(9.3e-04)	32.77(9.21)	3.8e-04	1.97(15.99)	9.0e-01	56.58(6.76)	6.0e-17
22	rs42221575	15	60250434	a/g	—	5.4e-19	+++(9.1e-02)	−34.78(13.33)	9.1e-03	−46.33(11.38)	4.7e-05	−52.00(6.68)	6.7e-15
23	rs381479851	16	1819787	a/g	*PLEKHA6*	9.7e-14	+++(6.1e-01)	35.49(9.14)	1.0e-04	71.05(35.70)	4.7e-02	63.95(10.46)	9.7e-10
24	rs41603770	16	28319407	t/c	—	1.9e-13	-+-(2.8e-10)	−1.05(9.10)	9.1e-01	14.91(18.88)	4.3e-01	−95.03(9.62)	5.4e-23
25	rs110127719	16	42843345	t/c	*DRAXIN*	1.6e-15	−−−(2.5e-03)	16.19(8.75)	6.4e-02	16.98(10.90)	1.2e-01	90.82(10.89)	7.3e-17
26	rs109008971	16	63549240	t/c	*KIAA1614*	9.8e-14	−−−(8.8e-03)	13.02(8.67)	1.3e-01	20.36(9.93)	4.0e-02	62.02(8.11)	2.1e-14
27	rs41666403	17	24539327	a/g	—	1.9e-15	−−−(1.9e-03)	20.08(9.63)	3.7e-02	13.88(11.93)	2.4e-01	68.53(8.19)	5.9e-17
28	rs41628722	17	60612635	a/c	*C17H12orf49*	1.6e-13	−−−(5.5e-02)	22.80(8.98)	1.1e-02	14.72(10.33)	1.5e-01	60.09(8.35)	6.3e-13
29	rs41915550	19	45164235	a/g	*GJC1*	2.2e-18	+++(2.2e-07)	10.21(8.54)	2.3e-01	6.55(10.77)	5.4e-01	85.13(8.29)	1.0e-24
30	rs109153933	19	54920324	t/c	—	8.1e-17	+++(4.0e-06)	3.32(10.98)	7.6e-01	24.51(10.19)	1.6e-02	64.95(6.91)	5.4e-21
31	rs436825729	20	32670639	a/g	*FBXO4*	4.0e-41	??-(1.0e+00)	9.33(290.50)	9.7e-01	−11.46(18.43)	5.3e-01	136.43(10.16)	4.0e-41
32	rs110840223	23	30279220	a/g	*ZKSCAN8*	3.2e-15	?++(4.1e-02)	−31.62(12.52)	1.2e-02	28.75(16.57)	8.3e-02	117.97(14.83)	1.8e-15
33	rs110836892	23	34231434	c/g	—	1.7e-12	+++(3.0e-01)	32.56(10.20)	1.4e-03	30.38(20.02)	1.3e-01	46.43(7.37)	3.0e-10
34	rs42094442	26	21800887	a/g	—	2.4e-11	−−−(4.9e-02)	−76.45(27.83)	6.0e-03	−48.60(10.00)	1.2e-06	−55.84(12.64)	1.0e-05
35	rs43060720	26	30146092	a/g	—	5.2e-15	+++(8.9e-02)	−18.91(8.83)	3.2e-02	−45.01(15.33)	3.3e-03	−109.33(15.04)	3.7e-13
36	rs41606803	26	44892777	t/c	*CTBP2*	2.3e-14	−−−(8.0e-04)	11.22(8.97)	2.1e-01	93.05(16.37)	1.3e-08	44.52(7.16)	5.1e-10

1 *Populations in order are MON, NOR, and HOL; “+” and “–” denote positive and negative substitution effects of the alternate allele. “?” indicates that the variant did not segregate in the respective population. The P-value of Cochran's Q-test for heterogeneity of the effect sizes across breeds is given in parentheses, and is significant if P < 0.05*.

**Table 2:** Lead SNP variants at 48 QTL for fat yield and percentage in milk.

								**MON**		**NOR**		**HOL**	
**QTL**	**Reference SNP cluster ID**	**CHR**	**BP**	**Allele**	**Nearby gene**	**P_meta_**	**Effect direction (and evidence of heterogeneity) across three populations^1^**	**b(se)**	***p***	**b(se)**	***p***	**b(se)**	***p***
1	rs42243014	2	14950298	t/c	*NEUROD1*	7.50e-14	+++(1.4e-01)	−0.15(0.03)	7.40e-08	−0.14(0.04)	1.90e-03	−0.13(0.03)	4.00e-06
2	rs110410153	2	26212397	t/c	*MYO3B*	2.40e-13	−−−(4.7e-01)	−0.07(0.03)	5.60e-03	−0.09(0.04)	1.00e-02	−0.14(0.02)	1.60e-10
3	rs29025329	2	44947955	a/g	*NMI*	2.10e-17	+++(3.4e-01)	−1.50(0.39)	1.50e-04	−1.09(0.49)	2.50e-02	−2.05(0.28)	1.30e-13
4	rs110253060	2	109873484	a/g	—	1.10e-16	+-+(3.9e-06)	1.42(0.48)	2.90e-03	−0.38(0.52)	4.70e-01	4.23(0.46)	4.40e-20
5	rs110900415	3	7416886	a/c	*NOS1AP*	4.20e-14	+++(2.6e-03)	−0.92(0.37)	1.30e-02	−0.25(0.47)	5.90e-01	−2.17(0.27)	2.60e-15
6	rs110824611	3	11040167	t/c	*OR6N2*	2.30e-29	+++(2.6e-01)	−0.16(0.03)	4.60e-09	−0.14(0.05)	4.10e-03	−0.25(0.03)	1.20e-20
7	rs42370628	3	63136013	t/c	—	1.10e-22	??+(1.0e+00)	−1.18(0.59)	4.40e-02	1.63(0.43)	1.60e-04	3.00(0.31)	1.10e-22
8	rs109919360	3	113845303	a/g	*USP40*	1.80e-12	+-+(1.6e-05)	−1.63(0.41)	6.50e-05	1.08(0.70)	1.20e-01	−2.17(0.30)	2.60e-13
9	rs209323908	5	88807577	a/c	*ABCC9*	5.60e-16	−−−(1.5e-01)	−2.55(0.46)	3.30e-08	−2.39(0.65)	2.20e-04	−1.99(0.40)	5.10e-07
10	rs41653559	5	94570828	a/g	—	3.50e-31	−−−(4.3e-07)	0.19(0.07)	4.00e-03	0.32(0.20)	1.10e-01	0.81(0.07)	3.20e-35
11	rs109168860	7	20325702	a/c	*KDM4B*	3.70e-16	−−−(4.6e-01)	0.09(0.03)	4.70e-04	0.10(0.04)	1.50e-02	0.14(0.02)	1.70e-12
12	rs110304043	7	45135080	t/c	*GRIN3B*	6.10e-15	−−−(1.9e-01)	−0.93(0.40)	1.80e-02	−1.72(0.50)	5.70e-04	−1.97(0.29)	8.00e-12
13	rs109939693	7	59590759	t/c	—	2.80e-17	−−−(4.6e-01)	−2.97(0.93)	1.40e-03	−1.69(0.48)	4.10e-04	−2.24(0.32)	1.20e-12
14	rs110116017	7	102889266	t/c	—	7.20e-10	-?-(1.5e-02)	−0.05(0.04)	1.80e-01	0.00(0.04)	9.20e-01	−0.15(0.02)	8.70e-11
15	rs42589512	8	31586227	c/g	*LURAP1L*	5.60e-15	+++(2.0e-01)	0.15(0.03)	2.40e-07	0.29(0.08)	2.00e-04	0.12(0.02)	1.10e-06
16	rs109004526	8	76473229	a/c	*AQP7*	1.50e-13	−−−(2.4e-02)	−0.68(0.40)	8.90e-02	−1.74(0.80)	2.90e-02	−3.49(0.47)	1.60e-13
17	rs41616289	8	93896073	a/g	—	1.10e-31	+++(2.4e-02)	0.10(0.03)	1.60e-04	0.17(0.04)	4.00e-05	0.25(0.02)	1.80e-26
18	rs41593345	9	45303296	t/g	*PREP*	4.30e-18	−−−(1.4e-03)	−0.04(0.03)	1.80e-01	−0.12(0.03)	5.90e-04	−0.18(0.02)	5.30e-18
19	rs41612115	9	51985736	t/c	—	5.10e-21	+++(1.4e-02)	1.36(0.46)	2.90e-03	1.48(0.65)	2.20e-02	3.32(0.36)	8.50e-20
20	rs41579916	9	79191994	c/g	—	3.80e-15	+++(3.8e-02)	−0.06(0.02)	1.60e-02	−0.13(0.07)	6.10e-02	−0.16(0.02)	1.50e-14
21	rs110588142	10	46388294	a/t	*HERC1*	2.40e-29	-++(8.8e-14)	−0.23(0.44)	6.00e-01	0.69(0.08)	7.00e-18	0.31(0.03)	3.30e-26
22	rs41612429	11	30220330	t/c	*FBX011*	1.40e-19	−−−(1.8e-01)	−0.14(0.02)	7.50e-10	−0.10(0.03)	4.70e-03	−0.14(0.02)	3.30e-10
23	rs41668653	11	63467507	t/c	*RAB1A*	7.50e-48	+++(1.6e-03)	0.11(0.03)	2.10e-05	0.24(0.04)	1.70e-11	0.30(0.02)	8.20e-37
24	rs110436636	11	103289035	a/g	—	4.10e-47	+-+(1.7e-55)	0.24(0.02)	2.00e-24	−0.31(0.03)	2.20e-19	0.34(0.02)	9.70e-62
25	rs109657055	12	69106382	t/c	—	1.60e-28	+-+(3.5e-06)	0.17(0.03)	7.80e-10	−0.01(0.04)	7.90e-01	0.21(0.02)	1.00e-25
26	rs108961732	13	46433697	t/c	*ADARB2*	5.50e-14	+-+(6.7e-06)	−0.15(0.02)	1.50e-10	0.04(0.04)	2.40e-01	−0.13(0.02)	7.30e-10
27	rs109421300	14	1801116	t/c	*DGAT1*	2.20e-284	−−−(6.1e-42)	15.24(1.95)	5.80e-15	9.12(0.84)	3.20e-27	12.77(0.35)	5.30e-287
28	rs109234250	14	1802265	a/g	*DGAT1*	2.6e-322	+++(6.3e-53)	4.13(0.13)	6.30e-234	2.57(0.06)	0.00e+00	2.82(0.03)	0.00e+00
29	rs41718954	14	21129363	a/g	*PRKDC*	1.30e-25	+++(2.6e-08)	0.04(0.02)	7.60e-02	0.04(0.03)	2.40e-01	0.26(0.02)	2.80e-32
30	rs109007040	14	67443766	t/c	*STK3*	1.90e-75	+++(1.2e-10)	−0.16(0.02)	2.20e-11	−0.09(0.04)	1.50e-02	−0.46(0.03)	2.40e-74
31	rs110249976	15	53166998	t/c	*FCHSD2*	1.90e-44	−−−(3.2e-09)	−0.34(0.06)	1.30e-07	−0.02(0.03)	5.20e-01	−0.47(0.03)	8.30e-47
32	rs42331218	15	62236286	t/c	—	4.10e-19	−−−(1.7e-01)	−1.61(0.54)	2.80e-03	−1.56(0.47)	8.70e-04	−2.46(0.31)	1.70e-15
33	rs378003729	16	24591986	t/c	—	3.90e-14	-+-(1.3e-04)	−2.47(0.37)	2.10e-11	0.07(0.55)	9.00e-01	−1.53(0.28)	4.00e-08
34	rs41628722	17	60612635	a/c	—	3.80e-16	-+-(6.5e-05)	1.09(0.38)	4.40e-03	−0.09(0.48)	8.50e-01	3.00(0.34)	1.40e-18
35	rs41874204	18	38471116	a/g	*ZFHX3*	4.90e-11	-+-(7.9e-07)	−0.10(0.45)	8.30e-01	0.04(0.47)	9.20e-01	−2.35(0.28)	3.10e-17
36	rs109882115	18	58067310	a/g	*ENSBTAG00000039491*	9.00e-28	+++(7.0e-03)	0.20(0.04)	1.50e-07	0.06(0.04)	1.30e-01	0.24(0.02)	2.20e-23
37	rs109751680	19	42976859	a/g	*STAT5B*	1.60e-37	-+-(2.5e-18)	0.08(0.03)	4.80e-03	−0.04(0.04)	2.50e-01	0.34(0.02)	3.50e-53
38	rs41921177	19	51326750	a/g	*CCDC57*	6.80e-25	−−−(1.2e-03)	5.26(0.87)	1.40e-09	0.58(0.91)	5.30e-01	2.69(0.30)	1.00e-19
39	rs207867568	20	31128620	t/c	*ENSBTAG00000033187*	6.60e-166	??+(1.0e+00)	−0.64(0.55)	2.40e-01	−0.01(0.04)	8.80e-01	−0.84(0.03)	6.60e-166
40	rs109262355	20	35249040	t/c	*FYB*	3.50e-16	−−−(1.3e-04)	−0.36(0.48)	4.50e-01	−1.50(0.58)	9.50e-03	−2.56(0.29)	1.60e-18
41	rs137102117	23	27450989	a/g	*LY6G5B*	4.20e-20	?+?(1.0e+00)	0.38(0.05)	7.60e-15	0.59(0.06)	4.20e-20	0.14(0.24)	5.60e-01
42	rs110212032	24	37652964	a/g	—	6.40e-19	+++(8.0e-04)	0.10(0.02)	3.80e-05	0.01(0.03)	8.40e-01	0.18(0.02)	2.60e-18
43	rs41659834	26	14814944	t/c	*MYOF*	1.30e-17	+++(4.3e-01)	−1.36(0.40)	6.30e-04	−1.99(0.47)	2.10e-05	−2.05(0.31)	2.10e-11
44	rs42090237	26	20290497	t/c	*GOT1*	3.00e-19	+++(2.0e-01)	0.08(0.03)	1.90e-03	0.24(0.05)	6.60e-06	0.15(0.02)	2.40e-13
45	rs109330072	26	32442734	t/g	—	5.10e-13	−−−(1.7e-01)	−4.00(1.01)	7.50e-05	−5.09(4.75)	2.80e-01	−2.54(0.41)	4.40e-10
46	rs109877780	27	26628005	t/g	—	1.90e-35	+++(4.7e-04)	−0.10(0.03)	9.30e-05	−0.13(0.04)	3.00e-03	−0.25(0.02)	1.50e-33
47	rs109599512	27	36117365	a/g	*GOLGA7*	2.00e-72	−−−(1.7e-03)	0.21(0.03)	9.30e-16	0.39(0.04)	8.70e-25	0.29(0.02)	4.50e-38
48	rs42181608	29	29254185	a/g	*PKNOX2*	3.70e-13	−−−(3.9e-02)	−0.62(0.41)	1.30e-01	−1.80(0.46)	1.10e-04	−1.95(0.30)	9.10e-11

1 *Populations in order are MON, NOR, and HOL; “+” and “–“ denote positive and negative substitution effects of the alternate allele. “?” indicates that the variant did not segregate in the respective population. The P-value of Cochran's Q-test for heterogeneity of the effect sizes across breeds is given in parentheses, and is significant if P < 0.05*.

**Table 3:** Lead SNP variants at 29 QTL for protein yield and percentage in milk.

								**MON**		**NOR**		**HOL**	
**QTL**	**Reference SNP cluster ID**	**CHR**	**BP**	**Allele**	**Nearby gene**	**P_meta_**	**Effect direction (and evidence of heterogeneity) across three populations^1^**	**b(se)**	***p***	**b(se)**	***p***	**b(se)**	***p***
1	rs29025329	2	44947955	a/g	*NMI*	2.94e-18	+++(9.6e-02)	−0.82(0.32)	1.0e-02	−1.28(0.36)	3.6e-04	−1.61(0.21)	4.8e-15
2	rs42889305	2	70680985	t/g	—	1.15e-18	−−−(3.2e-04)	0.36(0.33)	2.8e-01	1.52(0.43)	4.5e-04	1.84(0.20)	3.0e-19
3	rs110532777	3	21640254	t/c	*POLR3C*	8.05e-11	−−−(3.8e-02)	0.49(0.30)	1.0e-01	0.52(0.35)	1.5e-01	1.38(0.21)	3.3e-11
4	rs208464932	3	48855688	a/g	*SLC44A3*	1.17e-13	??+(1.0e+00)	—	—	—	—	2.02(0.27)	1.2e-13
5	rs42737927	3	113907407	t/c	*UGT1A1*	3.31e-12	+-+(4.3e-05)	−0.49(0.34)	1.4e-01	0.05(0.35)	8.9e-01	−1.73(0.21)	3.6e-16
6	rs43703011	6	87181619	t/g	*CSN2*	9.84e-31	−−−(6.4e-03)	−1.92(0.42)	5.9e-06	−3.71(0.51)	2.2e-13	−2.67(0.32)	1.2e-16
7	rs41609745	7	65358446	t/c	—	1.89e-14	+++(2.4e-04)	0.11(0.32)	7.3e-01	1.52(0.39)	8.6e-05	1.57(0.20)	1.0e-14
8	rs109205829	8	30088133	c/g	*NFIB*	1.79e-17	+++(3.2e-01)	1.22(0.32)	1.2e-04	1.69(0.36)	2.7e-06	1.29(0.21)	7.4e-10
9	rs42254183	8	65107050	a/g	—	5.25e-12	+++(1.2e-03)	−0.26(0.43)	5.5e-01	−1.13(0.53)	3.4e-02	−2.20(0.29)	6.7e-14
10	rs109630763	8	103526477	t/c	—	1.85e-12	-+-(1.5e-03)	−5.37(1.26)	2.0e-05	0.20(0.35)	5.6e-01	−1.79(0.27)	3.0e-11
11	rs41612115	9	51985736	t/c	—	1.64e-22	+++(4.8e-02)	1.32(0.37)	3.8e-04	1.19(0.48)	1.3e-02	2.46(0.27)	1.1e-19
12	rs41647455	10	15333419	a/g	—	1.22e-16	−−−(1.1e-07)	−0.32(0.46)	4.9e-01	−0.23(0.38)	5.3e-01	−2.12(0.21)	1.8e-23
13	rs43625971	10	47689396	t/c	*TLN2*	1.47e-13	+++(9.7e-03)	−0.36(0.32)	2.7e-01	−1.25(0.35)	3.7e-04	−1.70(0.24)	1.5e-12
14	rs109186903	10	94083525	t/c	—	2.51e-18	??+(1.0e+00)	—	—	—	—	2.23(0.26)	2.5e-18
15	rs41684057	13	17104043	t/c	*PRKCQ*	1.48e-10	+++(8.7e-02)	1.14(0.83)	1.7e-01	1.19(0.47)	1.1e-02	1.30(0.21)	8.6e-10
16	rs110991150	13	40815971	c/g	—	3.29e-20	+++(4.0e-02)	1.59(0.46)	5.1e-04	7.27(3.66)	4.7e-02	2.64(0.30)	4.1e-18
17	rs109421300	14	1801116	t/c	*HSF1*	8.03e-78	+++(8.2e-11)	−8.08(1.59)	3.7e-07	−2.70(0.62)	1.5e-05	−4.91(0.26)	3.1e-78
18	rs41617796	14	37670101	t/c	—	3.11e-11	+++(1.6e-01)	0.63(0.31)	4.0e-02	0.74(0.40)	6.2e-02	1.71(0.27)	2.4e-10
19	rs109020671	14	60893281	a/g	—	2.25e-12	+++(4.4e-01)	−0.81(0.33)	1.5e-02	−1.25(0.43)	3.8e-03	−1.29(0.21)	1.5e-09
20	rs42221575	15	60250434	a/g	—	2.21e-14	+++(1.4e-02)	−0.63(0.46)	1.7e-01	−1.28(0.39)	1.1e-03	−1.49(0.20)	1.7e-13
21	rs109053739	15	77590401	g/c,t	*CKAP5*	6.60e-13	+-+(3.1e-03)	−2.24(0.64)	4.6e-04	0.12(0.63)	8.4e-01	−1.93(0.27)	9.7e-13
22	rs41628722	17	60612635	a/c	—	1.48e-16	−−−(2.8e-03)	0.94(0.31)	2.7e-03	0.26(0.36)	4.7e-01	2.12(0.25)	4.7e-17
23	rs110031071	19	51767413	a/g	*PDE6G*	1.59e-09	−−−(5.7e-02)	−0.36(0.31)	2.3e-01	−1.34(0.36)	1.6e-04	−1.06(0.21)	2.6e-07
24	rs436825729	20	32670639	a/g	—	3.66e-304	??+(1.0e+00)	—	—	—	—	−0.59(0.01)	0.00
25	rs41592357	22	24219999	t/c	—	5.63e-16	−−−(4.3e-01)	−1.22(0.35)	4.6e-04	−1.50(0.35)	1.8e-05	−1.24(0.20)	1.5e-09
26	rs385583887	23	29349996	a/g	—	1.20e-17	+?+(1.2e-07)	0.08(0.31)	7.9e-01	—	—	2.81(0.28)	9.0e-24
27	rs41624917	26	15383866	a/g	*PLCE1*	2.09e-11	+++(5.0e-03)	0.87(0.39)	2.6e-02	0.14(0.51)	7.9e-01	1.46(0.21)	1.2e-12
28	rs109839618	26	42403875	a/g	*TACC2*	8.56e-13	+++(1.6e-02)	−0.64(0.31)	3.9e-02	−0.47(0.40)	2.4e-01	−1.91(0.26)	2.2e-13
29	rs41686102	29	41989397	t/c	*SLC22A6*	1.38e-51	+++(1.1e-16)	−0.03(0.02)	3.3e-02	−0.06(0.03)	3.0e-02	−2.3(0.01)	1.4e-65

1 *Populations in order are MON, NOR, and HOL; “+” and “–” denote positive and negative substitution effects of the alternate allele. “?” indicates that the variant did not segregate in the respective population. The P-value of Cochran's Q-test for heterogeneity of the effect sizes across breeds is given in parentheses, and is significant if P < 0.05*.

A QTL on BTA8 at 29–30 Mb with the same lead SNP at BTA8:30088133 (rs109205829) was observed in both Holstein and Montbeliarde. This lead SNP is an intronic variant within the *NFIB* gene, and the meta-analysis had a lower *P*-value (*P* = 2.3 × 10^−22^) than either of the single breed GWAS (*P* = 8.4 × 10^−14^). Another QTL on BTA22 at 23–26 Mb with a lead SNP at BTA22:24219999 (rs41592357) was observed in all three breeds. Rs41592357 is an intergenic variant 1.38 Mb upstream of the *CNTN6* gene and 0.65 Mb downstream of the *CNTN4* gene. Some QTL overlapped between Holstein and Montbeliarde, some with same lead SNP, but others with different lead SNP, e.g., BTA15 at 57–61 Mb and BTA25 at 10–11 Mb. The lead SNP for the QTL on BTA15 were rs110049689 (BTA15:57333896, *P* = 1.5 × 10^−7^, *MYO7A* gene) in Montbeliarde, and rs41690133 (BTA15:56842162, *P* = 2.4 × 10^−12^, intergenic) in Holstein. On BTA25, the lead SNP was rs42062121 (BTA25:11015593, *SNX29* gene) in both Holstein (*P* = 1.1 × 10^−7^) and Montbeliarde (*P* = 2.5 × 10^−6^).

##### Fat yield and percentage.

We observed QTL on 19 chromosomes where Holstein had 26 QTL, Montbeliarde had 12 QTL, Normande had 5 QTL. Meta-analysis confirmed BTA14:1802265 (*P* = 1.5 × 10^−760^, *DGAT1* gene). Other QTL lead SNP whose meta-analysis *P*-values exceeded the GWAS significance threshold include: rs110824611 (BTA3:11040167, *P* = 1.2 × 10^−7^, *OR6K6* gene), rs41616289 (BTA8:93896073, *P* = 2.9 × 10^−7^, *intergenic*), rs109394729 (BTA10:46486647, *P* = 1.9 × 10^−7^, *USP3* gene), rs41668653 (BTA11:63467507, *P* = 1.0 × 10^−10^, *intergenic*), rs110249976 (BTA15:53166998, *P* = 4.8 × 10^−9^, *FCHSD2* gene), rs41921177 (BTA19:51326750, *P* = 8.0 × 10^−14^, *CCDC57* gene), and rs109599512 (BTA27:36117365, *P* = 9.4 × 10^−17^, *GOLGA7* gene).

We observed QTL overlapping in Holstein and Montbeliarde and in Holstein and Normande, but with different lead SNP. The two lead SNP for QTL significantly associated in both Holstein and Montbeliarde were BTA12:15173534 (*P* = 3.1 × 10^−13^, *NUFIP1* gene, 14.5–15.6 Mb), and rs41921177 (BTA19:51326750, *P* = 1.1 × 10^−19^, *CCDC57* gene, 51–52 Mb). The three lead SNP for QTL significantly associated in both Holstein and Normande were rs109394729 (BTA10:46486647, *USP3* gene, 43–47 Mb), rs109599512 (BTA27:36117365, *GOLGA7* gene, and 35.5–38.5 Mb), and rs41668653 (BTA11:63467507, *intergenic*, 61.2–64.9 Mb).

##### Protein yield and percentage.

We observed QTL on 22 chromosomes where Holstein had 31 QTL, Montbeliarde had 18 QTL, Normande had 22 QTL. We confirmed already known causal variants, BTA6:87199843 (*P* = 9.5 × 10^−31^, *CSN2* gene), and BTA14:1802265 (*P* = 8.0 × 10^−78^, *DGAT1* gene). As presented in Table 4, new QTL lead SNP whose meta-analysis *P*-value exceeded the within breed GWAS significance levels included: rs41593345 (BTA9:45303296, *P* = 4.3 × 10^−18^, *PREP* gene), rs41668653 (BTA11:63467507, *P* = 7.5 × 10^−48^, *RAB1A* gene), and rs109882115 (BTA18:58067310, *P* = 9.0 × 10^−28^, *CEACAM18* gene). Two QTL overlaps were observed between Holstein and Montbeliarde, on BTA6 (85–88 Mb) with the most significant lead SNP at BTA6:87199843 (*HSTN*), and on BTA21 with lead SNP. rs29011638 (BTA21:37684315, *P* = 3.8 × 10^−7^, *intergenic*). We observed two QTL overlapping between Holstein and Normande on BTA7 (13–14 Mb) with the most significant SNP, rs41568613 (BTA7:13526016, *P* = 7.7 × 10^−6^, *intergenic*), and on BTA26 (21.5–24.5 Mb) with lead SNP, rs29014382 (BTA26:22339074, *P* = 1.8 × 10^−9^, *intergenic*). We observed two QTL overlapping among all three breeds on BTA16 (1–2 Mb) with lead SNP, rs42450080 (BTA16:1608132, *P* = 1.2 × 10^−10^, *intergenic*), and on BTA23 (50–51 Mb) with lead SNP, rs42507912 (BTA23:51118713, *P* = 1.3 × 10^−11^, *GMDS* gene). QTL overlap between fat and protein was evident, e.g., on BTA11 at 61–64 Mb with its lead SNP at rs41668653 (BTA11:63467507, *P* = 7.2 × 10^−13^, *intergenic*), and on BTA14 at 1.6–1.9 Mb with the most significant lead SNP being BTA14:1802265 (*P* = 2.6 × 10^−322^, *DGAT1* gene).

**Table 4:** Lead SNP variants at 13 QTL for success rate of insemination of lactating cows.

								**MON**		**NOR**		**HOL**	
**QTL**	**Reference SNP cluster ID**	**CHR**	**BP**	**Allele**	**Nearby gene**	**P_meta_**	**Effect direction (and evidence of heterogeneity) across three populations^1^**	**b(se)**	***p***	**b(se)**	***p***	**b(se)**	***p***
1	rs109193035	2	24147156	a/g	*ITGA6*	1.07e-06	+?+(3.3e-01)	0.02(0.01)	6.3e-02	0.01(0.01)	4.5e-01	0.07(0.02)	3.9e-06
2	rs29010833	2	66885868	t/g	—	1.07e-06	+++(5.5e-01)	−0.01(0.00)	1.2e-01	−0.01(0.00)	1.9e-02	−0.01(0.00)	3.5e-05
3	rs110027085	2	101307425	a/g	—	3.00e-07	+++(3.6e-01)	−0.01(0.00)	1.5e-01	−0.01(0.01)	5.9e-02	−0.02(0.00)	1.9e-06
4	rs41897801	8	23964538	a/g	*MLLT3*	6.39e-06	+++(1.7e-01)	0.01(0.00)	1.2e-02	0.00(0.00)	9.8e-01	0.01(0.00)	2.7e-05
5	rs42588799	8	59245157	a/c	—	6.07e-06	+++(6.5e-01)	−0.01(0.00)	9.5e-02	−0.01(0.01)	1.4e-02	−0.01(0.00)	4.0e-04
6	rs109483390	9	76868290	a/g	—	5.24e-10	+++(3.1e-01)	0.01(0.00)	2.9e-02	0.01(0.00)	8.6e-02	0.02(0.00)	8.3e-09
7	rs42412333	10	39278374	a/g	—	1.19e-08	+++(5.1e-01)	−0.01(0.01)	4.3e-02	−0.01(0.00)	2.7e-03	−0.01(0.00)	5.2e-06
8	rs109269005	11	67347867	a/g	*ANTXR1*	8.39e-07	−−−(2.9e-01)	0.01(0.00)	1.5e-02	0.01(0.02)	5.9e-01	0.04(0.01)	6.0e-06
9	rs41620105	11	82643114	t/c	—	3.93e-07	+++(2.0e-01)	0.00(0.00)	3.1e-01	−0.01(0.00)	1.4e-02	−0.01(0.00)	3.0e-06
10	rs110425867	14	8264685	t/c	*ZFAT*	1.63e-11	+++(3.2e-01)	−0.02(0.01)	2.8e-04	−0.01(0.01)	2.1e-01	−0.03(0.01)	9.9e-09
11	rs41568388	15	39634819	a/g	—	4.94e-07	+++(1.9e-02)	0.00(0.00)	5.0e-01	0.00(0.00)	4.1e-01	0.02(0.00)	1.5e-08
12	rs109269484	20	40301530	a/c	*ADAMTS12*	4.76e-05	+++(5.2e-01)	0.01(0.00)	2.3e-03	0.01(0.01)	1.7e-01	0.01(0.00)	9.7e-03
13	rs42049541	24	29349786	t/g	—	1.44e-06	+-+(3.8e-05)	0.00(0.00)	3.5e-01	−0.01(0.00)	1.5e-01	0.02(0.00)	1.8e-10

1 *Populations in order are MON, NOR, and HOL; “+” and “–” denote positive and negative substitution effects of the alternate allele. “?” indicates that the variant did not segregate in the respective population. The P-value of Cochran's Q-test for heterogeneity of the effect sizes across breeds is given in parentheses, and is significant if P < 0.05*.

#### Fertility

For the success rate at insemination of lactating cows, we observed QTL on 9 chromosomes where Holstein had 13 QTL, Montbeliarde had 2 QTL, Normande had 1 QTL. As presented in Table 5, the lead SNP for new QTL from meta-analysis that exceeded the genome wide threshold (*P* < 1.03 × 10^−6^) included rs109483390 (BTA9:76868290, *P* = 5.2 × 10^−10^, 0.1 Mb upstream of *TNFAIP3* gene and 0.07 Mb downstream of *PERP* gene), rs42412333 (BTA10:39278374, *P* = −1.2 × 10^−8^, 38–40 Mb, 0.45 Mb downstream of *RPL10L* gene), and rs110425867 (BTA14:8264685, *P* = 1.6 × 10^−11^, *ZFAT* gene 8.1–8.3 Mb). Lead SNP and QTL intervals for QTL observed only in Holstein were: rs41589904 (BTA8:76582220, *P* = 7.5 × 10^−9^, *UBE2R2* gene, 74–77 Mb), rs41593363 (BTA9:53806121, *P* = 1.3 × 10^−8^, 49–55 Mb, and 6.5 kb upstream of *GPR63* gene), rs110543856 (BTA18:48150900, *P* = 1.1 × 10^−18^, *SIPA1L3* gene, 43–48 Mb), rs110588160 (BTA21:47117318, *P* = 2.6 × 10^−14^, *intergenic*, 43–48 Mb), rs29023151 (BTA22:23303686, *P* = 1.6 × 10^−11^, *IL5RA* gene, 22–24 Mb), rs109629413 (BTA24:33624891, *P* = 7.1 × 10^−15^, *TMEM241* gene, 28–33 Mb), and two, QTL on BTA26: rs43158237 (BTA26:6741237, *P* = 2 × 10^−11^, *intergenic*, 6.7–6.9 Mb) and rs42096924 (BTA26:33443386, *P* = 2.8 × 10^−11^, *intergenic*, 24–33 Mb). Lead SNP and QTL intervals for QTL observed only in Montbeliarde were: rs41657531 (BTA9:39816061, 1.1 × 10^−8^, *RPF2* gene, 38–44 Mb), and rs42417896 (BTA11:34068419, 4.6 × 10^−6^, *intergenic*, 31–39 Mb). The suggestive fertility QTL observed in Normande was rs41570140 (BTA11:30065164, 1.6 × 10^−4^, *FBX011* gene, 27–30 Mb).

**Table 5:** Lead SNP variants at 16 QTL for stature.

								**MON**		**NOR**		**HOL**	
**QTL**	**Reference SNP cluster ID**	**CHR**	**BP**	**Allele**	**Nearby gene**	**P_meta_**	**Effect direction (and evidence of heterogeneity) across three populations^1^**	**b(se)**	***p***	**b(se)**	***p***	**b(se)**	***p***
1	rs41645172	2	109778246	t/c	—	3.3e-10	+++(8.1e-10)	0.31(0.04)	3.9e-17	0.22(0.07)	2.3e-03	0.18(0.01)	2.7e-01
2	rs43121344	7	68281468	t/c	—	7.6e-19	−−−(4.6e-01)	0.30(0.05)	4.5e-08	0.44(0.12)	3.0e-05	0.04(0.01)	1.1e-09
3	rs110652403	7	90460692	t/c	—	1.2e-11	−−−(1.3e-02)	−0.04(0.04)	3.0e-01	−0.26(0.01)	1.5e-02	−0.05(0.01)	4.8e-12
4	rs109703572	9	46739973	t/c	—	1.8e-23	+++(6.3e-03)	−0.09(0.04)	1.8e-02	−0.22(0.06)	7.7e-05	−0.06(0.01)	4.9e-21
5	rs41664341	9	80859295	a/g	—	5.8e-22	−−−(2.6e-02)	0.15(0.04)	5.2e-05	0.10(0.06)	8.7e-02	0.06(0.01)	2.5e-19
6	rs109860141	10	51558941	a/g	*FAM63B*	5.9e-16	+++(2.6e-09)	0.34(0.04)	2.4e-20	0.22(0.06)	4.2e-04	0.02(0.01)	7.9e-03
7	rs110128058	10	58318595	a/g	*GNB5*	3.5e-18	+++(3.5e-09)	0.40(0.04)	3.6e-22	0.22(0.07)	2.5e-03	0.04(0.01)	6.4e-04
8	rs43430263	12	53241975	t/g	*SLAIN1*	6.5e-16	-+-(1.4e-04)	0.19(0.04)	5.7e-07	−0.04(0.06)	4.6e-01	0.06(0.01)	3.3e-14
9	rs29019889	13	33366445	a/g	*NSUN6*	6.4e-16	+++(9.0e-03)	0.21(0.03)	4.5e-10	0.10(0.10)	3.3e-01	0.04(0.01)	3.5e-09
10	rs134215421	14	25006125	t/c	*PLAG1*	8.6e-140	-??(1.0e+00)	1.70(0.07)	8.6e-140	—	—	—	—
11	rs41584869	19	17569423	a/g	*SPACA3*	2.4e-11	−−−(1.3e-01)	0.32(0.06)	4.7e-07	0.18(0.07)	6.0e-03	0.03(0.01)	6.9e-05
12	rs110291686	19	43941099	a/c	*U2; ARLL4D*	1.7e-15	−−−(3.3e-01)	0.20(0.04)	1.4e-07	0.14(0.06)	1.7e-02	0.04(0.01)	1.3e-08
13	rs109082193	20	18413770	a/g	*ERCC8*	1.3e-12	+++(7.0e-01)	−0.19(0.05)	5.9e-04	−0.11(0.05)	3.9e-02	−0.05(0.01)	3.2e-09
14	rs385826566	20	39917111	a/c	*ADAMTS12*	5.0e-22	-??(1.0e+00)	−0.37(0.04)	5.0e-22	—	—	—	—
15	rs133559545	21	8262352	a/g	*IGF1R*	2.4e-17	+??(1.0e+00)	−0.30(0.04)	2.4e-17	—	—	—	—
16	rs41644660	26	19460476	t/c	—	1.1e-27	++?(3.2e-07)	−0.27(0.05)	3.5e-08	−0.76(0.07)	9.3e-27	—	—

1 *Populations in order are MON, NOR, and HOL; “+” and “–” denote positive and negative substitution effects of the alternate allele. “?” indicates that the variant did not segregate in the respective population. The P-value of Cochran's Q-test for heterogeneity of the effect sizes across breeds is given in parentheses, and is significant if P < 0.05*.

#### Stature

We identified 16 QTL on 11 chromosomes associated with stature in the three breeds (Table 5). The most significant variant was observed in the Montbeliarde breed and was BTA14:25006125 (*P* = 8.6 × 10^−140^ *PLAG1* gene). Seven novel QTL for stature displayed heterogeneity across the three breeds, e.g., rs43121344 (BTA7:68281468, intergenic and 0.14 Mb toward the 3′-flanking region of *MRPL22* gene) was lead SNP in all three breeds with *P*_NOR_ = 3.0 × 10^−4^, *P*_MON_ = 4.5 × 10^−8^, *P*_HOL_ = 1.1 × 10^−9^, and P_META_ = 7.6 × 10^−19^. Lead SNP for breed specific QTL in Montbeliarde include: rs41645172 (BTA2:109778246, *P* = 3.9 × 10^−17^, *DOCK10* gene), rs109860141 (BTA10:51558941, *P* = 2.4 × 10^−20^ *FAM63B* gene), BTA14:25006125 (*P* = 8.6 × 10^−140^, *PLAG1* gene), and BTA20:39917111 (*P* = 5.0 × 10^−22^, *ADAMTS12* gene). Lead SNP for QTL observed in Normande include rs41644660 (BTA26:19460476, *P* = 3.5 × 10^−8^, 73 kb downstream of *HPS1* gene). Two QTL lead SNP observed in Holstein include rs110652403 (BTA7:90460692, *P* = 4.8 × 10^−12^, 0.16 Mb upstream of *MEF2C* gene, and 0.3 Mb downstream of *TMEM161B* gene), and rs43430263 (BTA12:53241975, *P* = 3.3 × 10^−14^, *SLAIN1* gene).

### Meta-analysis heterogeneity among three breeds

**Figure 3:**
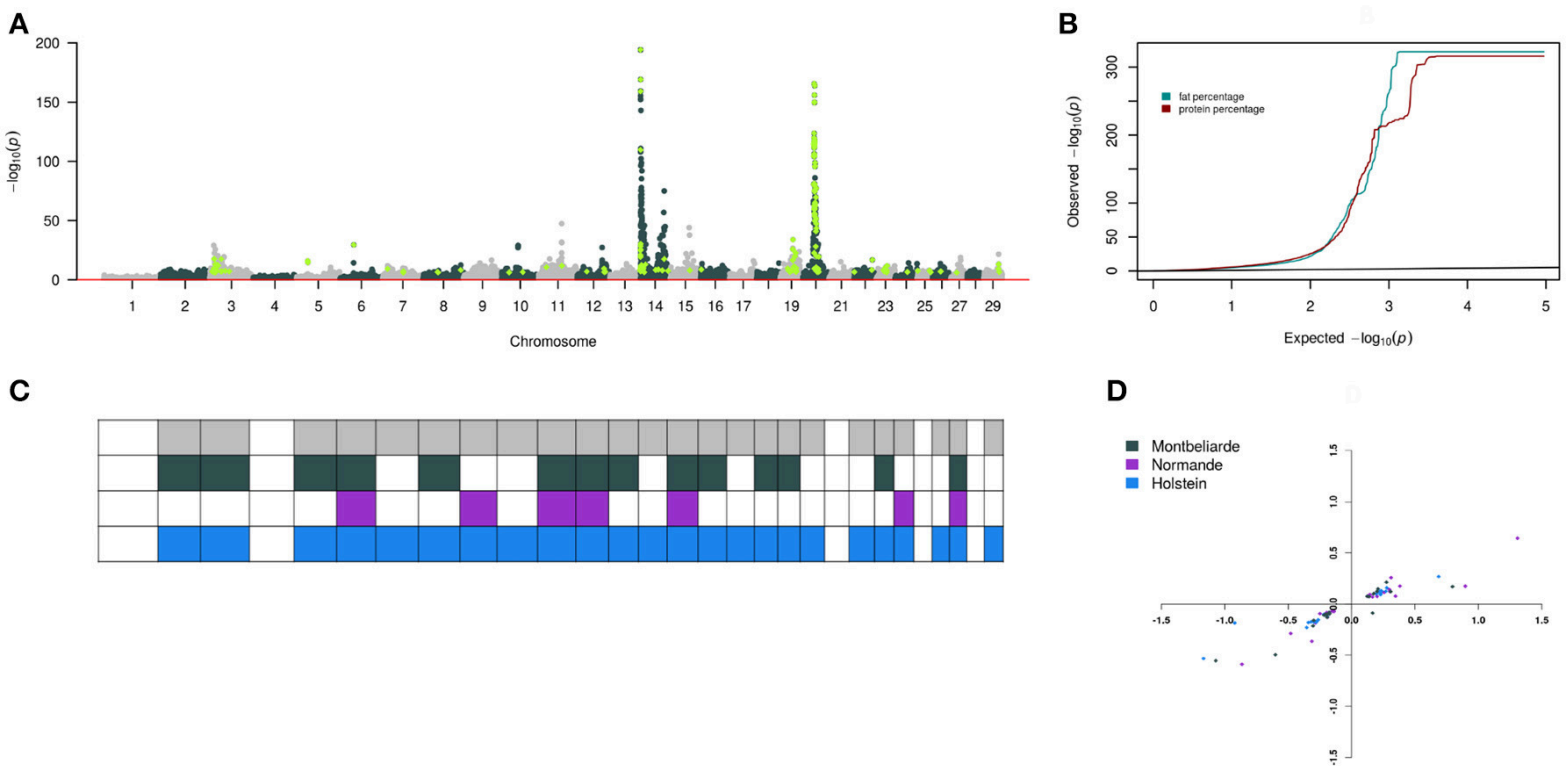
Heterogeneity of SNP association with fat and protein in milk across three cattle breeds. **(A)** Composite Manhattan plot that shows the association of 43,421 imputed variants including 5,609 pre-selected sequence variants with fat and protein in the meta-analysis. The composite Manhattan plot summarizes the results of the meta-analyses with each dot representing the more significant *P*-value that was observed across both traits. Light green represents sequence variants with *P* < 1 × 10^−6^. **(B)** Quantile-quantile plot of the meta-analyses. Green and brown color represent *P*-value of 43,421 imputed variants for fat and protein, respectively. **(C)** Overview of 59 QTL that were significant at *P* < 1 × 10^−6^ in the meta-analysis and within-breed association studies. Each column represents one of 29 *Bos taurus* autosomes. Row colors are breed specific with the top row being overall meta-analysis QTL. Filled squares indicate that QTL were significant in the respective breeds and chromosome. **(D)** Allelic substitution effects of 36 QTL on fat and protein standardized with the phenotypic standard deviations. The vertical axis is protein, and the horizontal axis is fat.

As an example of the heterogeneity observed from a meta-analysis of the three breeds, we observed peaks from a combined Manhattan plot for fat and protein percentage (Figures 3A,B) and standardized the allelic substitution effects by the phenotypic standard deviation of fat and protein percentage meta-analysis results. We observed heterogeneity (Cochran's Q, *P* < 0.05) for 36 QTL from the meta-analysis in three breeds (Figure 3D). We then overlay observed breed specific QTL for these two traits and Holstein had 26 QTL in 24 chromosomes, Montbeliarde had 17 QTL in 14 chromosomes, and Normande had 16 QTL in 7 chromosomes (Figure 3C).

## Discussion

Our meta-analysis of association summary statistics for seven traits across three French dairy cattle breeds discovered 120 QTL including 13 QTL that had not been detected at *P* < 1.03 × 10^−6^ in the within-breed analyses. In agreement with previous studies (Pausch et al., 2017) our results show that combining GWAS summary data from several breeds increases the power of association studies. However, for lowly heritable traits, and for distinct populations, a meta-analysis may not be as robust as the within-breed GWAS (Raven et al., 2014). For instance, within-breed GWAS showed associated SNP for fertility had no overlap between breeds probably due to small effects per locus (i.e., low power) and possibly the action of many, reasonably rare recessive lethal alleles. Holstein, with larger sample size, had associated SNP in strong LD and thus had stronger fertility predicted effects. In some instances, the meta-analysis increased the power, and consequently, the confidence interval for some fertility related QTL became visible, e.g., QTL on BTA2, 8 and 20 (Table 4). However, in other instances, the QTL detection power reduced when the QTL are private, e.g., the meta-analysis for fertility lost power compared to the within-breed GWAS when the Normande breed (with the smallest number of animals) was included in the analysis, e.g., QTL on BTA15 and 24 (Table 4).

One advantage of a meta-analysis over within-breed GWAS is that it is expected to result in more precision in QTL position due to the breakdown of long-range LD due to many generations of recombination since the breeds were separated (Raven et al., 2014; Pausch et al., 2016). In this study, we performed a within-breed GWAS first, followed by meta-analyses of GWAS summary statistics from three breeds using a weighted Z-score model to get the *P*-value for each SNP. In our study, the meta-analysis increased the *P*-value for some variants, probably because the QTL was not segregating across populations. Meta-analysis decreased *P*-value for some variants, and consequently, some QTL peaks became more distinct and narrower. For example, a region on BTA6:87296809 was strongly associated with protein yield and mapped near the protein-coding genes *ODAM* and *CSN3* in a 120 kb region. Bovine odontogenic, ameloblast-associated (*ODAM*) participates in structuring the extracellular matrix to attach epithelial cells to mineralized surfaces thus forming a protective seal that is antagonistic to bacterial invasion (Fouillen et al., 2017). On the other hand, Casein kappa *(CSN3)* is associated with daily milk yield (Bonfatti et al., 2011; Bartonova et al., 2012) as well as better cheese properties (Di Gregorio et al., 2017).

Strongly associated SNP (*P* < 1 × 10^−50^) were identified in all three French dairy breeds. Among these were several SNP that had previously been described such as those close to the DGAT1-K232A mutation on BTA14 (Grisart et al., 2002). These SNP were highly associated with all milk production traits in all three breeds. A very significant sequence variant at BTA5:93948357 (rs209372883) was associated with protein and fat in Holstein and Montbeliarde; it has previously been ascribed to a variant in *MGST1* (microsomal glutathione S-transferase 1; Raven et al., 2016), an upstream intron variant. *MGST1* is also an inflammation response gene which is highly expressed through pregnancy and lactation (Church et al., 2012). Previous studies in Japanese Black cattle (Wang et al., 2005) suggested upregulation of *MGST1* during adipocyte development in the longissimus muscle. Slightly upstream, a highly significant peak was centered within *SLC15A5*, a protein-coding gene. Our results suggest that both *MGST1* and *SLC15A5* may contain variants affecting milk production. Raven et al. (2014) identified a fat yield QTL within 3kbp of *MGST1* using a multi-breed analysis and we report a QTL which was within 2kbp of the genic region of *MGST1* using meta-analysis. Similar studies in Canadian Holstein, and using a similar density chip (50 k), reported a QTL 200 bp from that reported in our study (Li et al., 2010). The same Canadian Holstein study reported several fat yield candidate genes including *SLC2A3* and *GDF3* at 101.7–101.8 Mb and *LRP6, EMP1*, and *DUSP16* at 97–98 Mb on BTA5 (Li et al., 2010). Their results agrees with our meta-analysis results. Another very significant QTL for milk yield in Holstein was identified on BTA20 at ~38 Mb. The lead SNP of this QTL was located near the *PRLR* gene which has previously been associated with fertility in Holstein cows (Leyva-Corona et al., 2018; Marete et al., 2018b). The majority of the associated SNP with fertility, a low heritability trait, were 50 k chip variants. For instance, rs110992367, the lead SNP for a 3.3 Mb QTL for the success rate of insemination in Holstein cows was 0.38 Mb from the 5′ flanking region of the *G2E3* gene. Meta-analysis heterogeneity for this QTL was observed in all three breeds (*P* = 9.5 × 10^−3^), but opposite in the effect direction between Holstein and the other two breeds. The heterogeneity is important because the *G2E3* gene is an essential protein-coding gene that prevents apoptotic death during embryonic development (Brooks et al., 2008). This gene has been linked to apoptosis and cellular stress in US beef cattle (Howard et al., 2014) and somatic cell score in Chinese Holstein (Wang et al., 2015). This gene is a good candidate affecting cow fertility.

All QTL identified by meta-analysis were not significant in all within-breed analysis (Tables 1–6, Figure 3C) probably due to low power to reach significance at *P* < 1 × 10^−6^ or lack of segregation in the breed (Sham and Purcell, 2014). For instance, the variant mapping to the *PLAG1* gene only segregated in the Montbeliarde breed. However, most of the SNP had an effect in the same direction in all breeds for the more significant variants, e.g., BTA18:58067310 (*CEACAM18* gene, fat) and BTA10:51558941 (*FAM63B* gene, stature). This suggests that most detected QTL segregate in all three breeds even though they were not significant in the within-breed analysis. In this study, pre-selected sequence variants were directly genotyped or imputed from a large reference population genotyped with the EuroG10k chip, and thus were known with very high accuracy. This was evident from the high average allelic correlation (*r*^2^ = 0.97) between real and imputed genotypes for the three breeds. In conclusion, many QTL segregated across the three French cattle breeds more so for highly heritable traits. The meta-analysis across the breeds using the within-breed association summary data increased the power of QTL detection. This QTL information can be incorporated in routine genomic evaluation in French populations. If an independent population of dairy cattle is genotyped with the EuroG10k chip, then the true causal variant underlying the QTL may be validated in independent population/breed. The next step of this study is to use independent Nordic cattle data to validate the QTL reported here.

## Author contributions

AM analyzed data and wrote the manuscript. BG contributed to the design of the custom part of the EuroG10k chip, interpretation of results and revision of the manuscript. ML worked on the study design, secured project funding and revised the manuscript. SF contributed to the design of the custom part of the EuroG10k chip and the management of the marker information. GS interpreted the results and revised the manuscript. DB worked on the study design, interpretation of results and revision of the manuscript.

### Conflict of interest statement

The authors declare that the research was conducted in the absence of any commercial or financial relationships that could be construed as a potential conflict of interest.
