# Supplementary material for: A Meta-Analysis Including Pre-selected Sequence Variants Associated With Seven Traits in Three French Dairy Cattle Populations
Source: Front Genet. 2018 Nov 6;9:522. doi: 10.3389/fgene.2018.00522 (PMC6232291; doi:10.3389/fgene.2018.00522)
Supplement: Supplementary file 1 [file Table_1.DOCX]

| **Supplementary Table1: Number of animals genotyped with different Chips** | | | | |
| --- | --- | --- | --- | --- |
| Chip | MON | NOR | HOL | **Total** |
| 50k | 34,567 | 17,653 | 99,535 | **151,755** |
| LD | 8,674 | 4,858 | 25,108 | **38,640** |
| EuroGV1 | 6,288 | 2,715 | 11,933 | **20,936** |
| EuroGV2 | 8,065 | 2,292 | 9,962 | **20,319** |
| EuroGV3 | 16,990 | 5,822 | 27,810 | **50,622** |
| EuroGV4 | 25,155 | 10,918 | 46,298 | **82,371** |
| **Total** | **99,739** | **44,258** | **220,646** | **364,643** |
